# Dipeptidyl peptidase 4 inhibitor reduces tumor-associated macrophages and enhances anti-PD-L1-mediated tumor suppression in non-small cell lung cancer

**DOI:** 10.1007/s12094-023-03187-5

**Published:** 2023-04-28

**Authors:** Bei Zuo, Tao Li, Xiaoyun Liu, Shuling Wang, Jianxiang Cheng, Xiangqun Liu, Wenjie Cui, Hengliang Shi, Chunhua Ling

**Affiliations:** 1 Department of Respiratory and Critical Care Medicine, The First Affiliated Hospital of Soochow University, Suzhou, 215007 China; 2 Central Laboratory, The Affiliated Hospital of Xuzhou Medical University, Xuzhou, 221002 China; 3 Institute of Digestive Diseases, Xuzhou Medical University, Xuzhou, 221002 China; 4 Department of Respiratory and Critical Care Medicine, The Municipal Hospital Affiliated to Xuzhou Medical University, Xuzhou, 221116 China; 5 Department of Obstetrics and Gynecology, The Affiliated Hospital of Xuzhou Medical University, Xuzhou, 221004 China

**Keywords:** Non-small cell lung cancer, Tumor microenvironment, PD-L1, DPP4 inhibitor, Tumor-associated macrophages

## Abstract

**Purpose:**

The efficacy of immune checkpoint inhibitors such as programmed cell death ligand 1 (PD-L1) antibodies in non-small cell lung cancer (NSCLC) is limited, and combined use with other therapies is recommended. Dipeptidyl peptidase 4 (DPP4) inhibitors, a class of small molecule inhibitors, are highly effective for treating type 2 diabetes. Emerging evidence implicates DPP4 inhibitors as immunomodulators that modify aspects of innate and adaptive immunity. We evaluated the combination of a DPP4 inhibitor (anagliptin) and PD-L1 blockade in an NSCLC mouse model.

**Methods:**

The effect of the combination of anti-PD-L1 and anagliptin was evaluated in subcutaneous mouse models of NSCLC. Tumor-infiltrating immune cells were analyzed by flow cytometry. Bone marrow-derived monocytes of C57BL/6 mice were isolated in vitro to examine the underlying mechanism of anagliptin on the differentiation and polarization of macrophage.

**Results:**

Anagliptin dramatically improved the efficacy of PD-L1 antibody monotherapy by inhibiting macrophage formation and M2 polarization in the tumor microenvironment. Mechanistically, anagliptin suppressed the production of reactive oxygen species in bone marrow monocytes by inhibiting NOX1 and NOX2 expression induced by macrophage colony-stimulating factor, reduced late ERK signaling pathway activation, and inhibited monocyte-macrophage differentiation. However, the inhibitory effect was reactivated by lipopolysaccharide and interferon-gamma interacting with corresponding receptors during M1 macrophage polarization, but not M2.

**Conclusions:**

Anagliptin can enhance PD-L1 blockade efficacy in NSCLC by inhibiting macrophage differentiation and M2 macrophage polarization, and combination therapy may be a promising strategy for treating PD-L1 blockade therapy-resistant patients with NSCLC.

**Supplementary Information:**

The online version contains supplementary material available at 10.1007/s12094-023-03187-5.

## Introduction

Immune checkpoint inhibitors (ICIs) targeting the programmed death 1 (PD-1)/programmed cell death ligand 1 (PD-L1) pathway have been widely used and significantly improve overall survival in non-small cell lung cancer (NSCLC) [1]. However, they are only effective in a small proportion of patients, and the therapeutic efficacy is often hampered by M2 tumor-associated macrophages (TAMs) within the tumor microenvironment (TME). M2-type TAMs suppress anti-tumor immune responses and promote tumor growth by releasing anti-inflammatory cytokines and angiogenic factors [2–4]. Therefore, novel therapeutics targeting crucial signaling pathways that regulate the recruitment, polarization, and metabolism of TAMs during tumor progression can indirectly stimulate the recruitment and activation of cytotoxic T cells, which can synergize with ICIs [5, 6].

In early tumorigenesis, circulating monocytes migrate to tumor tissues under the action of chemokines secreted by tumor cells and related stromal cells, and then differentiate into macrophages by macrophage colony-stimulating factor (M-CSF) secreted by tumor cells [7]. Macrophages are generally categorized into two functionally contrasting subtypes: classically activated M1 macrophages and alternatively activated M2 macrophages. Activated by interferon-gamma (IFN-γ), microbial products (e.g., lipopolysaccharides [LPS]), or granulocyte–macrophage-CSF, M1-polarized macrophages express high levels of proinflammatory cytokines and are strongly microbicidal and antitumoral. On the other hand, macrophages were polarized into M2-type by Th2 cytokines such as interleukin (IL)-4, IL-10, and IL-13 and produce anti-inflammatory cytokines such as IL-10 and transforming growth factor beta (TGF-β) to promote tumor development; these are considered pro-tumor or “bad” macrophages [8, 9].

Previous research has shown that reactive oxygen species (ROS) is critical for macrophage differentiation and M2 polarization [10]. NADPH oxidases (NOXs) mediate the production of superoxides, which are the main intracellular ROS of non-mitochondrial origin and are related to the differentiation process of various cell types [11, 12]. NOX1 and NOX2 are the main subtypes of NOXs in bone marrow-derived monocytes (BMMs) and bone marrow-derived macrophages (BMDMs), and complete deletion of NOX1 and NOX2 results in significantly reduced ROS production in BMMs and inhibited macrophage differentiation and M2 polarization [13]. Therefore, it is speculated that targeted inhibition of NOX1 and NOX2 could alleviate immunosuppressive tumor microenvironments, which further improve the antitumor effect of ICIs.

Dipeptidyl peptidase 4 (DPP4), originally known as CD26, is expressed in almost all organs and tissues in the body. It is also widely expressed in vascular endothelial and epithelial cells and immune cells such as T cells, activated B cells, activated natural killer (NK) cells, dendritic cells, and macrophages [14]. DPP4 can enzymatically truncate proteins containing either L-proline or L-alanine at the penultimate position and has nearly 50 peptide substrates, including neuropeptide Y, substance P, and a variety of chemokines, which participate in several physiological and pathological processes and can promote or inhibit different types of cancer [15–17].

DPP4 has been shown to regulate C-X-C motif chemokine ligand 10 (CXCL10)-mediated lymphocyte migration in mouse melanoma- and hepatocellular carcinoma-transplanted tumors [18, 19]. DPP4 inhibitors preserve the activity of CXCL10 and make biologically active CXCL10 interact with its ligand (CXCR3) on NK and T cells, thereby increasing the chemotaxis of NK and T cells and further inhibiting tumor growth. Meanwhile, several studies have shown that DPP4 inhibitors exhibit anti-inflammatory and antioxidant effects in multiple diseases by modulating different subtypes of NOXs in various tissues [20–22]. Considering the immunomodulatory and antioxidant effects of DPP4 inhibitors, whether DPP4 inhibitors can potentiate the anti-tumor effect of ICIs in NSCLC remains unclear.

This study aimed to assess the effect of the combination of a DPP4 inhibitor (anagliptin) and PD-L1 blockade on NSCLC in a syngeneic mouse model. BMMs from C57BL/6 mice were isolated to observe the effect and intrinsic mechanism of anagliptin on the differentiation and polarization of macrophage.

## Methods

### Cell lines and treatments

Lewis lung cancer (LLC) cell line was purchased from KeyGEN BioTECH (Jiangsu, China). Cells were cultured in high-glucose DMEM (Gibco) medium supplemented with 10% heat-inactivated fetal bovine serum (Gibco), penicillin (100 units/ml, Gibco) and streptomycin (100 μg/ml, Gibco) in a humidified atmosphere of 5% CO_2_ air at 37 °C.

### Syngeneic tumor model

Six-week-old male C57BL/6 mice were subcutaneously injected in the flank with Lewis lung cancer (LLC) cells (1 × 10^6^ in 200 μL phosphate buffered saline [PBS]). The mice were either fed a diet containing anagliptin (2 g/kg) from the third day after the cell line injection; intraperitoneally injected with PD-L1 antibody (200 μg/head) twice weekly from 1 week after the cell line injection; intraperitoneally injected with PD-L1 antibody (200 μg/head) and simultaneously administered a diet containing anagliptin (2 g/kg); or fed a control diet, for 15–18 days. Tumor size (V) was measured every 3 days and calculated using the following formula: V(mm^3^) = width (mm)^2^ × length (mm) / 2. Five mice in each group were sacrificed at 15–18 days by carbon dioxide asphyxiation and tumor tissues were collected for flow cytometry, quantitative reverse transcription polymerase chain reaction (qRT-PCR), immunofluorescence, and western blotting.

### Protein expression analysis

Samples were homogenized in lysis buffer (pH 7.4) containing a protease inhibitor cocktail (HY-K0010, MedChenExpress), phosphatase inhibitor cocktail III (HY-K0023, MedChenExpress), 50 mM Tris, 150 mM NaCl, 5 mM ethylenediaminetetraacetic acid (EDTA), and 0.5% NP-40 (Sigma-Aldrich). The protein concentration was determined using a BCA Protein Assay kit (Tiangen Biotech). Sodium dodecyl-sulfate polyacrylamide gel electrophoresis (SDS-PAGE) was performed, and the samples were blotted onto a polyvinylidene fluoride (PVDF) membrane. Antibodies against DPP4 (ab28340, Abcam), F4/80 (28463-1-AP, Proteintech), CD206 (18704-1-AP, Proteintech), p44/42 MAPK (Erk1/2) (137F5) rabbit mAb (4695, Cell Signaling Technology), Phospho-p44/42 MAPK (Erk1/2) (Thr202/Tyr204) (9101, Cell Signaling Technology), signal transducer and activator of transcription 6 (STAT6) (ab32520, Abcam), and Phospho-STAT6 (phospho Y641) (ab263947, Abcam) were used at a dilution of 1:2000. Secondary antibody binding and detection were performed according to standard protocols using an enhanced chemiluminescence (ECL) detection reagent (Bio-Rad).

### Immunofluorescence analysis

Tumor tissues were fixed, embedded in paraffin, and sliced into 10-μm sections. Slices were routinely dewaxed and hydrated. Tris–EDTA buffer solution was used for antigen retrieval, 3% H_2_O_2_ was used for inactivation of endogenous peroxidase, and normal goat serum was used for blocking. The slides were then incubated overnight with F4/80 (ab6640, Abcam) and CD206 (ab64693, Abcam). Next, the membranes were incubated with horseradish peroxidase (HRP)-labelled goat anti-rabbit secondary antibodies in combination with 4′,6-diamidino-2-phenylindole (DAPI) for 1 h at room temperature. Finally, tissue immunofluorescence was observed using a fluorescence microscope (Olympus).

### RNA isolation and real-time PCR

Total ribonucleic acid (RNA) was extracted with TRIzol according to the manufacturer (15596026, Invitrogen) guidelines. Any remaining deoxyribonucleic acid (DNA) was removed by DNase I (RNase-Free) (EN0521, Invitrogen), and reverse transcription was carried out using the HiScript qRT SuperMix (+ gDNA wiper) kit (R123-01, Vazyme). RT-PCR for the genes of interest was performed using SYBR green (ChamQ SYBR Qpcr Master Mix) (Q311-02/03, Vazyme) on a LightCycler® 480 System (Roche). RT-PCR analysis confirmed the identity of the products using the melting curve analysis. The ratio of the amount of target messenger RNA (mRNA) to the amount of internal standard (β-actin) mRNA was determined as an arbitrary unit. Sequences of the primers used in this study are shown in Table 1.

**Table 1:** Sequences of the primers used in this study

Gene	Primer (5’–3’)
*Mrc-1*	F: CCT ATG AAA ATT GGG CTT ACG G
	R: CTG ACA AAT CCA GTT GTT GAG G
*Arg-1*	F: CAT ATC TGC CAA AGA CAT CGT G
	R: GAC ATC AAA GCT CAG GTG AAT C
*IL-10*	F: AAA CAA CTC CTT GGA AAA CCT CG
	R: TCC CCA ATG GAA ACA GCT TAA AC
*iNOS*	F: CTG CAG CAC TTG GAT CAG GAA CCT G
	R: GGG AGT AGC CTG TGT GCA CCT GGA A
*TNF-α*	F: GCT CTT CTG TCT ACT GAA CTT CGG
	R: ATG ATC TGA GTG TGA GGG TCT GG
*IL-12*	F: AGT GAC ATG TGG AAT GGC GT
	R: CAG TTC AAT GGG CAG GGT CT
*NOX1*	F: GTG CCT TTG CCT GGT TCA ACA AC
	R: AGC CAG TGA GGA AGA GAC GGT AG
*NOX2*	F: GAC AGG AAC CTC ACT TTC CAT A
	R: TGA AGA GAT GTG CAA TTG TGT G

*ARG-1* arginase-1; *IL* interleukin; *iNOS*, nitric oxide synthase; *MRC-1* mannose receptor C-type 1; *NOX* NADPH oxidase; *TNF-α* tumor necrosis factor-α

### Tissue cells preparation and flow cytometry analysis

Tumor tissue was minced into thin pieces and dissociated in DNase I (200 μg/mL) and collagenase I (1 mg/mL) in Dulbecco’s modified eagle medium (DMEM). Tissues were incubated for 1 h at 37 ℃ and gently stirred every 10 min. The reaction was stopped by adding 1 mL of 1 M EDTA, and the cells were filtered through a 70-μm cell strainer (BD Biosciences) and centrifuged at 1300 rpm at 4 ℃. Red blood cells were lysed with ACK (ammonium-chloride-potassium) lysis buffer on ice for 10 min. Cells (1 × 10^6^) were suspended in 100 μL of PBS + 0.02% EDTA and incubated with FVS780 to label the dead cells. Anti-mouse CD16/32 antibody (553141, BD Biosciences) was added to the samples at a 1:50 dilution for 20 min at 4 ℃ to block non-specific Fc receptor binding. Samples were then washed with fluorescent activated cell sorting (FACS) buffer (0.5% fetal bovine serum [FBS] in PBS) and stained with anti-CD45-PerCP (561047, BD Biosciences), anti-CD3-FITC (100203, BioLegend), anti-CD4-PE-CY7 (561099, BD Biosciences), anti-CD8-Percp (561092, BD Biosciences), anti-NK1.1-APC (561117, BD Biosciences), anti-CD11b-FITC (101,205, BioLegend), anti-F4/80-PE-CY7 (25–4801-82, eBioscience), and anti-CD86-APC (105011, BioLegend) antibodies at 4 ℃ for 20 min in the dark. For CD206 staining, samples were fixed and permeabilized with a fixation and permeabilization buffer set (88–8824-00, eBioscience) and then stained with anti-CD206-PE (12–2061-80, eBioscience) antibodies for 20 min in the dark. The samples were washed and resuspended in FACS buffer for sorting. The samples were sorted on a FACS Canto II sorter (BD Bioscience) equipped with Summit software. Flow cytometry data were analyzed using FlowJo X software.

### M1/M2 macrophage differentiation in vitro

To prepare BMDMs, the C57BL/6 mice were euthanized by CO_2_ asphyxiation and disinfected with 70% ethanol. Bone marrow mononuclear cells (BM-MNCs) were isolated from mouse femora and tibiae using a bone marrow mononuclear isolation kit (P6900, Solarbio, China). To achieve the simple purification of monocytes, BM-MNCs were cultured for 2 h. The attached monocyte-enriched cells were cultured in DMEM supplemented with 20% FBS, 2 mM glutamine, penicillin (100 units/mL), and streptomycin (100 μg/mL). For macrophage differentiation, monocytes were treated with anagliptin (50 μmol/L or 100 μmol/L) for 24 h, and then were cultured in the presence of M-CSF (20 ng/mL) to yield BMDMs, medium was changed on days 3 and 5 after plating, and the fully differentiated macrophages were treated on day 6. For M1/M2 polarization, BMDMs were treated by LPS (100 ng/mL) and INF-γ (20 ng/mL) for 24 h to generate M1 macrophages or by IL-4 (25 ng/mL) for 24 h to generate M2 macrophages. Polarized cells were collected for qRT-PCR and flow cytometry.

### CCK-8 assay

Cell proliferation and toxicity assays were performed using the cell counting kit (CCK)-8 assay (KeyGEN Biotech, Jiangsu, China). Approximately 1 × 10^4^ bone marrow-derived monocytes were seeded in 96-well plates, with 100 μL of medium in each well. After 24 h of cultivation, different doses of anagliptin were added, and the cells were incubated for 24 h. Each well was incubated with 10 μg of CCK-8 solution for 1–4 h away from light before the absorbance was measured at 450 nm using a Multiskan FC Microplate Reader (Thermo Scientific, USA).

### Generation of TAMs in vitro

Mouse Lewis lung cancer (LLC) cell line was cultured in DMEM (high glucose) medium supplemented with 10% fetal bovine serum (FBS) and 1% penicillin/streptomycin. When the LLC cells (4 × 10^5^) seeded in 6-well plates reached at least 80% confluence, 1 mL fresh medium was added to renew the medium. Following 48 h of culture, the medium was centrifuged at 500 g for 5 min and the supernatant was collected and referred to simply as CM. For induction of TAMs, mouse BMDMs which were pretreated or not with anagliptin (50 μmol/L or 100 μmol/L) were seeded in 12-well plates and stimulated with 1 ml mixture of supernatant and FBS-containing medium (1:1) for 48 h. The proportion of F4/80^+^CD206^+^ cells were measured by flow cytometry.

### In vitro T cell activation assay and co-culture system

To prepare T cells, C57BL/6 mouse spleens were ground with syringes, washed with PBS, and then passed through 70 μm cell strainers to gain single-cell suspensions. Red blood cells were lysed by ACK (ammonium-chloride-potassium) lysis buffer. Splenocytes were further separated with C57BL/6 mouse spleen lymphocyte separation solution (P8860, Solarbio) to obtain spleen lymphocytes. Obtained spleen lymphocytes were cultured in complete RPMI 1640 medium. For T-cell activation assays, anti-CD3e (5 μg/ml, 16-0031-82, eBioscience) diluted with PBS was pre-coated in 96 well plates overnight at 4 °C. The spleen lymphocytes cells were adjusted to 2 × 10^6^ cells/ml with complete RPMI and 100 ml cell suspension were added per well, then anti-CD28 (16-0281-82, eBioscience) was added to medium to a final concentration of 200 ng/well. For co-culture assay, TAMs at indicated ratios were added to the medium after T cell activation. After 4 days, Flow cytometry was used to measure the proportion of activated CD8^+^ T cells (IFN-γ^+^ CD8^+^ T cells)(anti-IFN-γ-PE, 505807, BioLegend)( anti-CD8-Percp, 561092, BD Biosciences).

### ROS detection

The Reactive Oxygen Species assay kit (CA1410-100 T, Solarbio, China) was used to measure the intracellular production of ROS. Fluorescence-free dichloro-dihydro-fluorescein diacetate (DCFH-DA) reagent (final concentration 10 μmol/L) was diluted in a serum-free medium at a ratio of 1:1000. Bone marrow-derived monocytes were treated with different concentrations of anagliptin for 24 h and then were collected and suspended in DCFH-DA. The cells were then incubated for 30 min in a 37 ℃ cell culture incubator. The cell suspensions were mixed every 5 min so that the probe was in full contact with the cells. After incubation, the cells were washed with serum-free cell culture, and M-CSF (20 ng/mL) was added at different time points. The percentage of DCFDA-positive cells was quantified using flow cytometry.

### Statistical analysis

Data from the three independent experiments are expressed as the mean ± standard deviation, unless otherwise described. Statistical analyses were performed using GraphPad Prism, version 8.0. Two-way analysis of variance (ANOVA) followed by Tukey’s test was used to determine the statistical significance level of the tumor size in multiple groups of mice at different time points. One-way ANOVA followed by Sidak's test was used to determine the statistical significance level in multiple groups. Statistical significance was set at *P* < 0.05.

## Results

### Anagliptin enhances anti-PD-L1-mediated tumor suppression

**Fig. 1:**
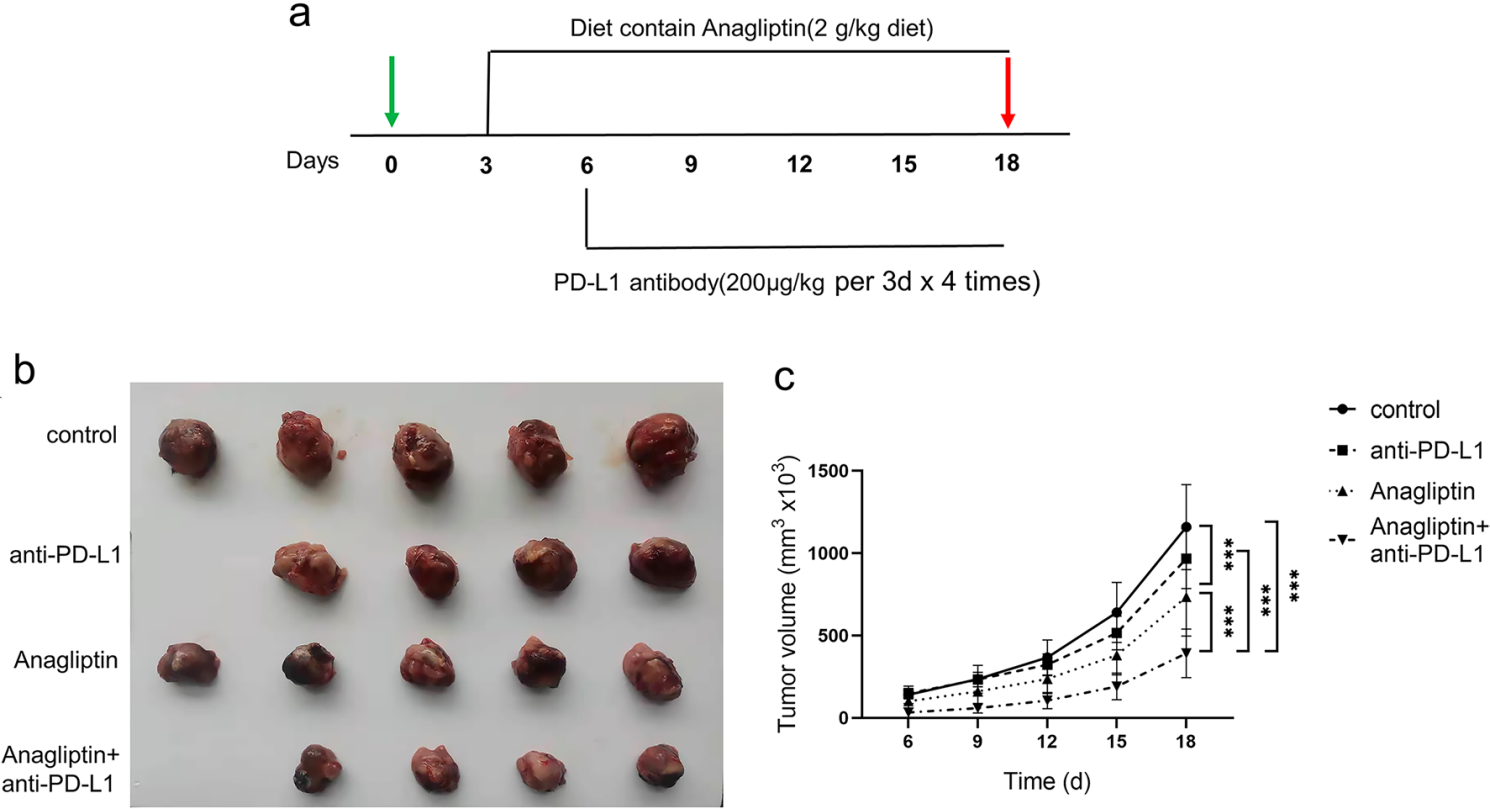
Anagliptin enhances anti-programmed cell death ligand 1 (PD-L1)-mediated Lewis lung cancer (LLC) tumor suppression. **a**, Schematic of anagliptin and anti-PD-L1 treatments. **b**, Representative image of LLC subcutaneous tumors in each group. **c**, The volume of subcutaneous LLC tumors from each group for the indicated times (mean ± S.E., n = 5/group). *, *P* < 0.05; ***, *P* < 0.01

To confirm whether DPP4 inhibitor, anagliptin, could potentiate the anti-tumor effect of PD-L1 blockade therapy, a syngeneic animal model was established in C57BL/6 mice by subcutaneous injection of murine Lewis lung cancer

(LLC) cells, which are poorly immunogenic. The mice with transplanted tumors were divided into four groups: (1) control group; (2) anagliptin group; (3) anti-PD-L1 antibody group; and (4) anagliptin + anti-PD-L1 antibody group (Fig. 1a). Anti-PD-L1 treatment alone had no significant inhibitory effect on the growth of tumors in mice, and the tumors in mice fed a diet containing anagliptin were significantly reduced compared to those in the normal diet group (Fig. 1b). The growth curve indicated that the tumors in the combined treatment group grew more slowly than those in the anagliptin or anti-PD-L1 antibody monotherapy groups (Fig. 1c).

### Anagliptin inhibits the formation of TAMs in the TME

**Fig. 2:**
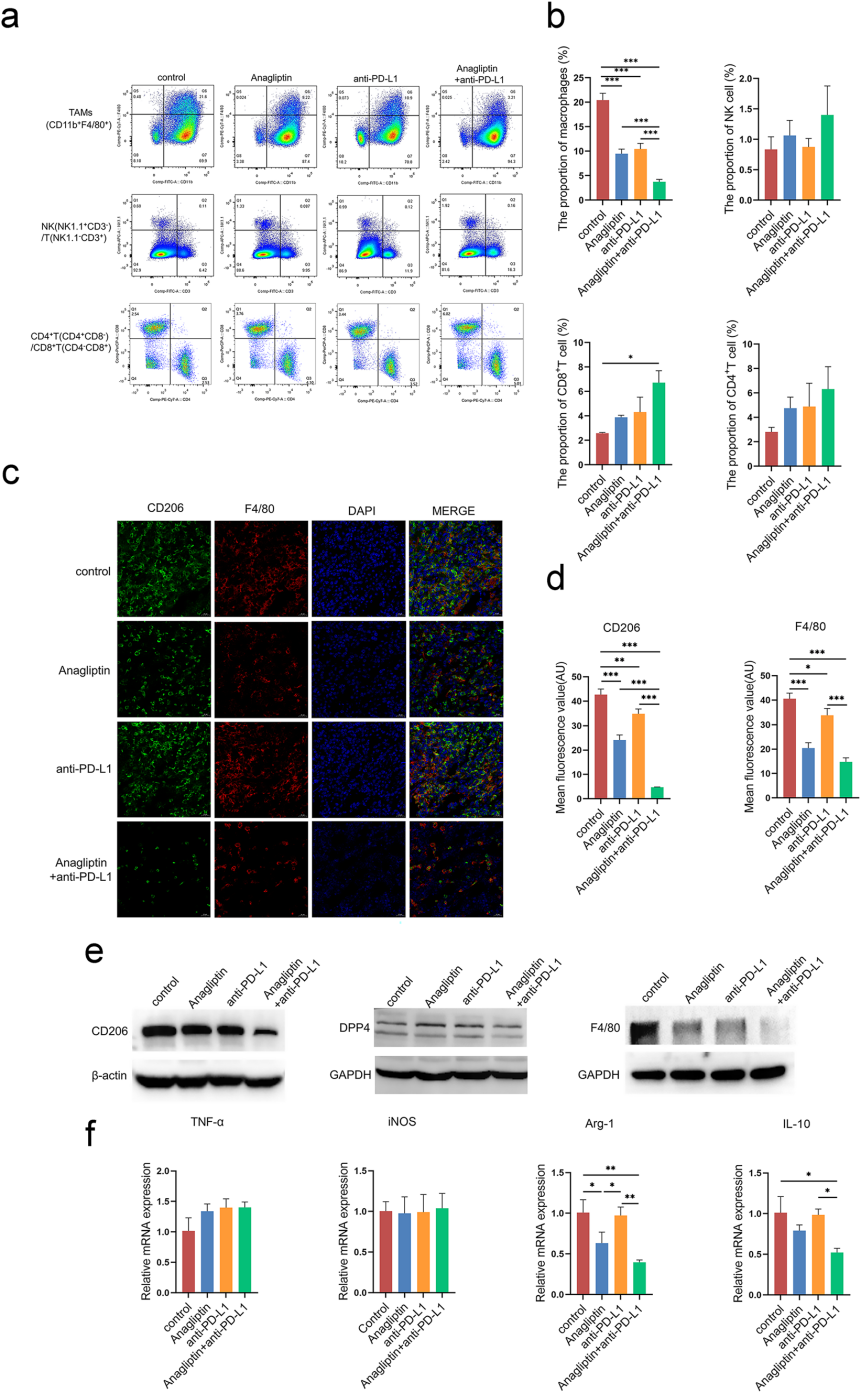
Anagliptin inhibits tumor-associated macrophages (TAMs) formation in the tumor microenvironment. **a, b**, The percentages of macrophages (CD11b^+^F4/80^+^), natural killer (NK) (NK1.1^+^CD3^−^) cells, CD4^+^T (CD3^+^CD4^+^CD8^−^) cells, and CD8^+^T (CD3^+^CD8^+^CD4^−^) cells in primary tumors on day 18 are determined by flow cytometry. ***, *P* < 0.01. **c, d**, Representative immunofluorescence images showing CD206 (green), F4/80 (red), and nucleus (blue) staining of the primary tumors from all groups on day 18. *, *P* < 0.05; **, *P* < 0.01. **e**, The expression levels of dipeptidyl peptidase 4 (DPP4), F4/80, and CD206 in primary tumors on day 18 from all groups are analyzed by western blotting. **f**, The expression levels of M1 cytokines (nitric oxide synthase [iNOS], tumor necrosis factor-α [TNF-α]) and M2 cytokines (interleukin 10 [IL-10], arginase-1 [ARG-1]) in primary tumors on day 18 from all groups are analyzed by real-time polymerase chain reaction. *, *P* < 0.05; **, *P* < 0.01

We investigated the effect of anagliptin on immune cell subsets in the subcutaneous transplanted tumor by flow cytometry. Compared to the control group, both anagliptin and anti-PD-L1 alone reduced the proportion of macrophages in tumor tissues, while combination treatment further enhanced this effect. Interestingly, anti-PD-L1 monotherapy did not significantly increase the infiltration of CD8^+^ T cells in tumor tissues, while the proportion of CD8^+^ T cells in the combined treatment group was significantly higher than that in the control group. Meanwhile, no significant differences in CD4^+^ T cells and NK cells were found between the groups. (Fig. 2a and b). Immunofluorescence and western blotting were performed to analyze macrophage typing in the TME. Anagliptin did not affect the expression of DPP4 in tumor tissues; both anti-PD-L1 and anagliptin alone could reduce the expression of macrophage-specific markers, F4/80, and the M2 macrophage-specific marker, CD206, in tumor tissues, while combined treatment significantly downregulated their expression compared to anti-PD-L1 treatment alone (Fig. 2c–e). Real-time qRT-PCR results showed significantly lower levels of the M2-related markers, arginase-1 (ARG-1) and IL-10, in the combined treatment group than in the control or anti-PD-L1 treatment groups. The expression levels of the M1-related markers, nitric oxide synthase (iNOS) and tumor necrosis factor-α (TNF-α), did not differ significantly between the groups (Fig. 2f).

### Anagliptin inhibits the differentiation and M2 polarization of BMDMs in vitro

**Fig. 3:**
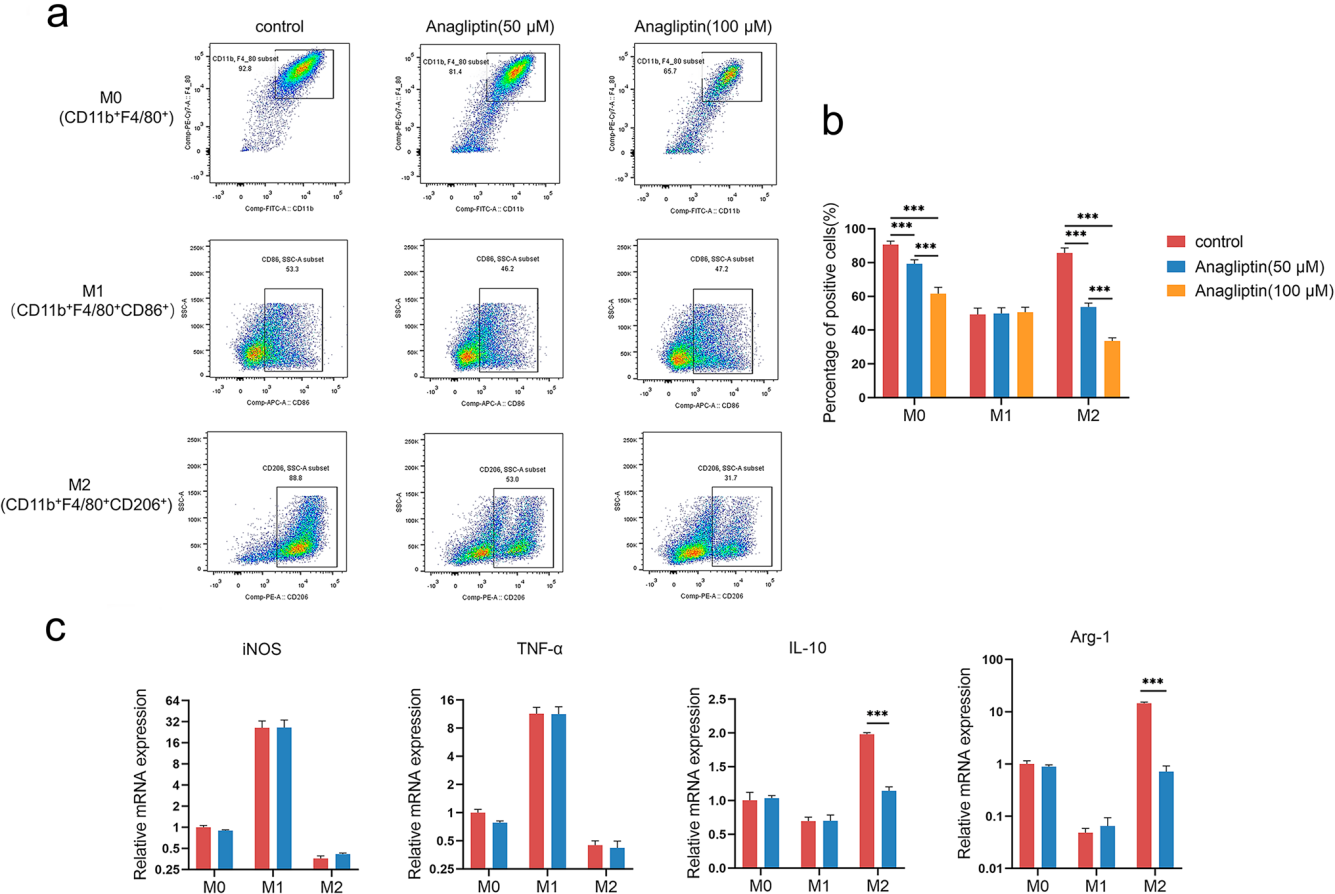
Anagliptin inhibits the differentiation and M2 polarization of mouse bone marrow-derived macrophages (BMDMs) in vitro. **a, b**, Flow cytometry analysis of the M0 marker (CD11b^+^F4/80^+^), M1 marker CD86, and M2 marker CD206 with anti-CD11b, anti-F4/80, anti-CD86, and anti-CD206 antibodies, respectively. ***, *P* < 0.01. **c**, Detection of M1 (nitric oxide synthase [iNOS], tumor necrosis factor-α [TNF-α]) and M2 (interleukin 10 [IL-10], arginase-1 [ARG-1]) markers by real-time polymerase chain reaction in the cells. ***, *P* < 0.01

BMMs of C57BL/6 mice were extracted and isolated in vitro to investigate the effect of anagliptin on the formation and polarization of TAMs. DPP4 was confirmed to be expressed in monocytes (Supplementary information, Fig. S1a), and CCK-8 assays showed that anagliptin (< 100 μM for 24 h) had no significant effect on BMMs viability (Supplementary information, Fig. S1b). To investigate the effect of anagliptin on the differentiation and polarization of macrophages, BMMs were dealed with anagliptin for a period of time before M-CSF induction, and then were first differentiated into BMDMs and further were perturbed to generate M1 and M2 populations. FACS analysis showed that the CD11b^+^F4/80^+^ BMDM population decreased significantly in the anagliptin treatment group in a concentration-dependent manner. When BMDMs were further polarized to M1 or M2 macrophages, the M1 population was similar between the groups, whereas the M2 populations which expressed specific marker CD206 were substantially reduced in a concentration-dependent manner in the anagliptin treatment group (Fig. 3a and b). qRT-PCR results showed no difference in the expression of the M1-specific marker iNOS and cytokine TNF-α between the groups; however, the levels of the M2-specific marker ARG-1 and cytokine IL-10 were significantly decreased in the anagliptin treatment group when macrophages were polarized toward M2 macrophages (Fig. 3c).

### Anagliptin inhibits the generation of TAMs and attenuates CD8^+^ T cells exhaustion in vitro

**Fig. 4:**
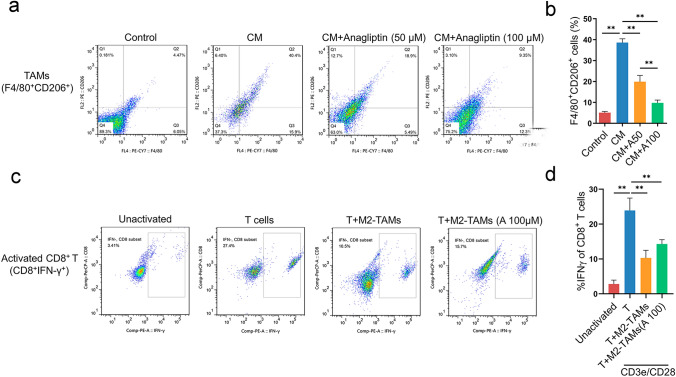
Anagliptin inhibits the generation of tumor-associated macrophages (TAMs) and attenuates CD8^+^ T cell exhaustion in vitro. **a, b**, BMDMs were polarized into M2 macrophages by LLC cells culture medium to imitate the formation of M2-type TAMs in TME. Flow cytometry analysis of the M2-TAMs marker (F4/80^+^CD206^+^) with anti-F4/80 and anti-CD206 antibodies, respectively. **, P < 0.01. **c, d**, Co-cultured the actived splenic T lymphocytes of C57BL/6 mice and TAMs which derived from the BMDMs. The amount of interferon-gamma (IFN-γ) in CD8^+^ T cells was detected by flow cytometry. **, P < 0.01

LLC cells culture medium significantly increased the proportion of F4/80^+^CD206^+^ cells in BMDMs, suggesting that LLC cells could secrete soluble factors to polarize BMDMs into M2-type macrophages which is the main phenotype of TAMs, and anagliptin could inhibit this effect in a concentration-dependent manner (Fig. 4a). It is well known that the antitumor effect of PD-L1 blockade is mainly mediated by CD8^+ ^T cells. To explore how anagliptin affects T cell immune function, we co-cultured the actived splenic T lymphocytes of C57BL/6 mice and TAMs which derived from the BMDMs. It was noticeable that TAMs inhibited the amount of IFN-γ in CD8^+^ T cells, and anagliptin mitigated the inhibitory effect of TAMs on CD8^+^T cells (Fig. 4b). The results above indicated that anagliptin targeting TAMs can attenuates the exhaustion of CD8^+^ T cells mediated by TAMs, and enhance the efficacy of PD-L1 blockade therapy.

### Inhibiting NOX1 and NOX2 by Anagliptin results in reduced ROS production during macrophage differentiation

**Fig. 5:**
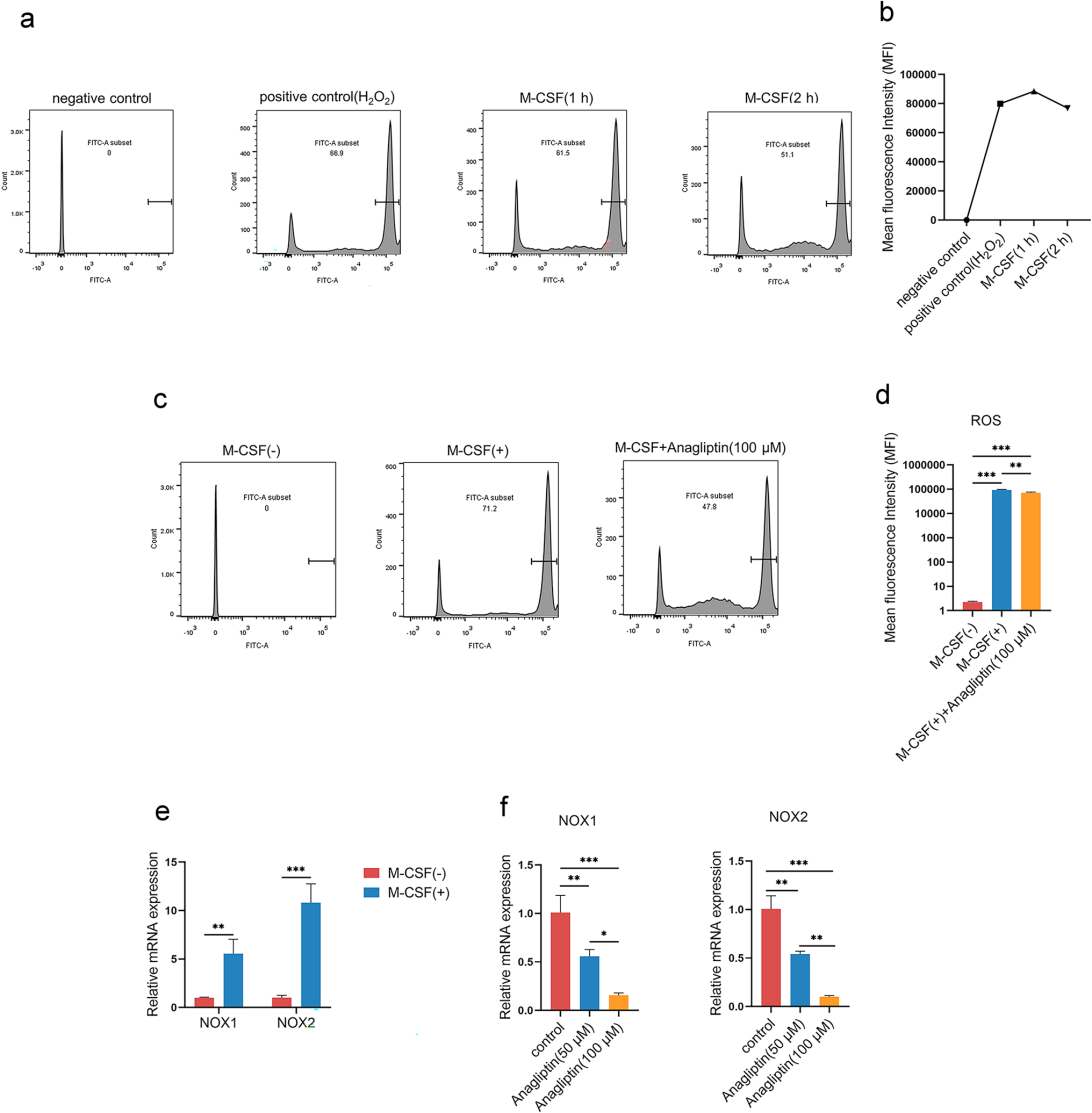
Inhibiting NOX1 and NOX2 by anagliptin results in reduced ROS production during macrophage differentiation. **a, b**, BMMs were either untreated or treated with M-CSF for the indicated time and collected for ROS measurement by flow cytometry. **c, d**, BMMs were either untreated or pretreated with anagliptin (100 µM) for 24 h. Cells were then treated with M-CSF for 1 h and collected for ROS measurement by flow cytometry. ROS production quantified by mean fluorescence intensity is shown. **e**, BMMs were either untreated or treated with M-CSF for 24 h, and the RNA of all cells was collected and extracted. **, *P* < 0.01. **f**, BMMs were either untreated or pretreated with anagliptin (50 μM or 100 μM) for 24 h before M-CSF induction, then M-CSF was added for induction for 24 h, and the RNA of all cells was collected and extracted. *, *P* < 0.05; **, *P* < 0.01

Considering that ROS play a critical role in the differentiation and M2 polarization of macrophages, we measured ROS production by dichloro-dihydro-fluorescein diacetate (DCFH-DA) assay. When M-CSF was applied to BMMs, a large amount of ROS was generated, and ROS production peaked at approximately 1 h and then gradually decreased (Fig. 5a and b). The intracellular ROS level of BMMs induced by M-CSF was significantly decreased by anagliptin pretreatment (Fig. 5c and d). In consideration of NOX1 and NOX2 are predominately responsible for ROS production in BMMs treated with M-CSF during macrophage differentiation, we measured the expression of NOX1 and NOX2 by qRT-PCR. Results displayed the expression levels of NOX1 and NOX2 were significantly increased after M-CSF induction in BMMs but decreased in anagliptin-treated cells (Fig. 5e and f).

### Anagliptin blocks macrophage differentiation and M2 macrophages polarization by inhibiting ERK pathway

**Fig. 6:**
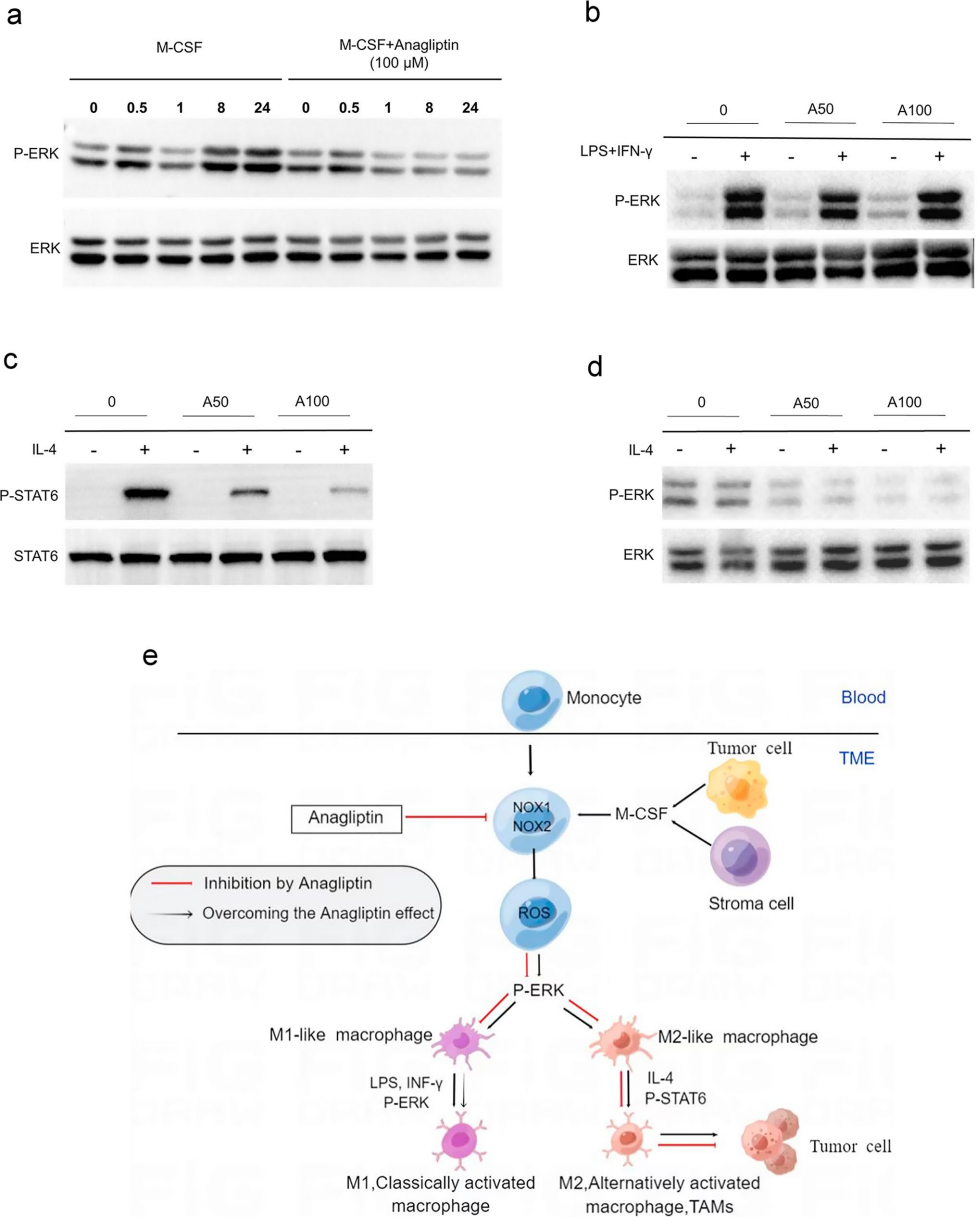
Anagliptin inhibits macrophage differentiation and M2 macrophage polarization by inhibiting late-phase extracellular signal-regulated kinase (ERK) pathway activation. **a**, Bone marrow-derived mononuclear cells (BMMs) were pretreated with or without anagliptin (100 μM) for 24 h and then treated with macrophage colony-stimulating factor (M-CSF) for the indicated times. The expression levels of p-ERK and ERK were determined by western blotting with the indicated antibodies. **b–d**, BMMs were either untreated or pretreated with anagliptin (50 µM or 100 µM) for 24 h and then differentiated for 6 days with M-CSF. On day 6, M-CSF-treated cells were treated with lipopolysaccharide (LPS) (100 ng/mL) and interferon-gamma (IFN-γ) (20 ng/mL) for 24 h or with interleukin (IL)-4 (25 ng/mL) for 24 h. The expression levels of p-ERK, ERK, P-signal transducer and activator of transcription 6 (p-STAT6), and STAT6 were determined by western blotting with the indicated antibodies. **e**, Schematic model illustrating the role of anagliptin in the monocyte-macrophage differentiation and M2 macrophage polarization

Since ROS-mediated activation of the MAP kinase, ERK, is involved in macrophage differentiation [23], we investigated ERK activation during macrophage differentiation. After M-CSF was added to BMMs, biphasic activation of the ERK pathway was observed, and late-phase ERK activation was significantly reduced in anagliptin-pretreated BMMs (Fig. 6a). These data imply that anagliptin inhibits macrophage differentiation by inhibiting late-phase ERK activation. We further investigated the mechanism by which anagliptin affected the M2 but not M1 macrophage polarization. LPS/INF-γ treatment induced ERK activation in all groups during the M1 polarization of macrophage, which may compensate for ERK activation defect in monocyte-macrophage differentiation, and further M1 polarization of these cells was not significantly affected (Fig. 6b). However, the M2 macrophage polarization was different. Previous studies have shown that the M2 polarization of macrophages induced by IL-4 was achieved by activating the Stat6 pathway, but not the ERK pathway. Consistent with this, our results showed that unobvious activation of ERK was found during M2 macrophage polarization, and activation of Stat6 which was mediated by IL-4 was severely impaired in anagliptin-treated macrophages. (Fig. 6c and d). Therefore, monocyte-macrophage differentiation and M2 macrophage polarization were significantly affected (Fig. 6e).

## Discussion

Immunotherapy strategies targeting the PD-L1 pathway have achieved remarkable success in treating NSCLC. Compared to traditional therapy, PD-L1 blockade significantly prolongs survival without obvious side effects in advanced NSCLC [24]. However, owing to tumor heterogeneity and individual immune system differences, PD-L1 blockade therapy is only effective in a small number of patients, while many patients show resistance to it. Thus, it is recommended that PD-L1 blockade in combination with other therapies [25]. In our study, we found that the DPP4 inhibitor, anagliptin, combined with anti-PD-L1 antibody enhanced the tumor-suppressive effect of PD-L1 blockade on NSCLC. In view of the important regulatory role of DPP4 inhibitors in the TME, we speculate that this type of drugs has great potential in tumor immunotherapy.

Previous studies have reported that DPP4 inhibitors can inhibit different types of tumors by preventing the hydrolysis of various chemokines. Da Silva et al. found that DPP4 inhibitors regulated CXCL10-mediated T-lymphocyte and NK cell migration in mouse melanoma- and hepatocellular carcinoma-transplanted tumor models [19]. In addition, they subsequently demonstrated that sitagliptin, a DPP4 inhibitor, exerted an anti-tumor effect by increasing CC motif chemokine ligand 11 (CCL11)-mediated eosinophil chemotaxis in syngeneic mouse models of liver and breast cancer, and combining sitagliptin with the inhibition of PD-1 and CTLA-4 significantly suppressed tumors expressing IL-33 [26]. In this study, murine LLC cells, which are poorly immunogenic, were subcutaneously injected into C57BL/6 mice to establish a syngeneic animal model. The results showed that anagliptin significantly enhanced anti-PD-L1-mediated tumor suppression, and combination therapy significantly inhibited tumor growth compared to monotherapy. By analyzing the infiltrating immune cells and the inflammatory immune profiles of tumors, we discovered that anagliptin reduced the proportion of macrophages and the M2/M1 macrophage ratio in the TME, meanwhile, the density of CD8^+^ T cells infiltrated to tumor was significantly higher in the combinated group compared with the control group. It is well known that anti-PD-L1 regresses the tumor growth is mainly mediated by CD8^+^ T cells, and M2 macrophages could hamper CD8^+^ T cell both infiltration and activity. Therefore, it is speculated that anagliptin enhanced the anti-tumor effect of anti-PD-L1 by decreased generation of M2 macrophages and indirectly enhanced infiltration and activity of CD8^+^ T cells.

Previous studies have confirmed that increased M2 macrophages in the TME correlate with poor clinical prognosis in various human cancers [27–30]. ICIs therapy may be hampered by M2 macrophages in the TME by several mechanisms, including: (1) M2 macrophage-derived cytokines downregulate major histocompatibility complex class II molecules in TAMs, resulting in diminished TH1 differentiation, which causes decreased anti-tumor activity [31]; (2) M2 macrophages inhibit anti-tumor immunity by suppressing CD8^+^ T-cell infiltration into the TME, which is accomplished via the down-regulation of CXCL9 and CXCL10 production by TAMs [32]; (3) T-cell cytotoxicity can be directly inhibited by M2 macrophage-mediated depletion of L-arginine and tryptophan [33]; (4) TAMs express ICI ligands, such as PD-L1. In an ICI therapy setting, such TAM ligands compete with tumor cell ligands, which directly inhibit cytotoxic T-cell functions [34]. In view of the immunosuppressive role of M2 macrophages in the TME, targeting TAM formation and various aspects of M2 macrophages polarization can enhance the efficacy of PD-L1 blockade therapy [2, 35–37].

Growing evidence shows that tumor cells can induce M2 polarization of TAMs through a variety of soluble factors, such as lactic acid, TGF-β, IL-10 and some exosomes [38–40]. In our study, BMDMs were polarized into M2 macrophages by LLC cells culture medium to imitate the formation of M2-type TAMs in TME and results displayed that anagliptin could inhibit the effect in a concentration-dependent manner. In order to further confirm that inhibition of M2 macrophage polarization achieved by anagliptin can enhance the anti-tumor effect of PD-L1 blockade, we co-cultured the actived splenic T lymphocytes of C57BL/6 mice and M2-type TAMs which derived from the BMDMs in vitro. It was noticeable that M2-type TAMs inhibited the amount of IFN-γ in CD8^+^ T cells, and anagliptin could mitigate the inhibitory effect. The results above indicated that anagliptin targeting the generation and M2 polarization of TAMs can attenuates the exhaustion of CD8^+^ T cells, and therefore enhance the efficacy of PD-L1 blockade therapy. To date, no studies exist on the relationship between DPP4 inhibitors and TAMs; therefore, we further investigated the role of DPP4 inhibitor in the formation and polarization of TAMs in vitro.

Consistent with the in vivo results, anagliptin-pretreated monocytes displayed impaired monocyte-macrophage differentiation and M2 macrophage polarization, but M1 polarization was not significantly affected. Zhang et al. discovered that ROS production was critical for macrophage differentiation and M2 polarization [10], and DPP4 inhibitors also exhibit similar anti-inflammatory and antioxidant effects in multiple diseases [20–22]. Therefore, we examined the effect of anagliptin on ROS production in monocytes induced by M-CSF. Our results showed that the intracellular ROS level of BMMs induced by M-CSF was significantly decreased by anagliptin pretreatment. Intracellular ROS of non-mitochondrial origin are mainly mediated by NOXs, and deletion of both NOX1 and NOX2 led to a dramatic reduced ROS production in macrophages and resulted in impaired monocyte-to-macrophage differentiation and M2 macrophage polarization [13]. DPP4 inhibitors also affect the expression of different subtypes of NOX in various tissues [41, 42]. We further investigated the effects of anagliptin on the expression of NOX1 and NOX2 in monocytes, and results showed that their expression levels in BMMs were significantly increased after M-CSF induction and dramatically decreased with anagliptin treatment, indicating that anagliptin inhibited ROS production in BMMs by inhibiting the expression of NOX1 and NOX2.

The MAP kinases, ERK and JNK, are activated and involved in macrophage differentiation [19], but only ERK activation is affected by ROS inhibition [10]. We further investigated the role of anagliptin in signaling pathway related to macrophage differentiation and polarization. The ERK pathway was biphasic activated in BMMs by the stimulation of M-CSF during macrophage differentiation, while only late-phase ERK activation was significantly decreased in anagliptin-pretreated BMMs. The important role of ERK activation in monocyte-macrophage differentiation is supported by experiments using the ERK inhibitor, U0126 [13]. Next, we investigated the effect of anagliptin on the signaling pathway related to M1 and M2 macrophage polarization. LPS/INF-γ binds to toll-like receptors on the surface of macrophages to activate ERK, which can compensate for the defect of ERK activation in monocyte-to-macrophage differentiation, and further polarized of these cells towards M1 macrophages was not affected. However, the Stat6 rather than ERK pathway was activated when macrophages were polarized to M2 macrophages, which could not compensate for the previous defect in ERK activation which was blocked by anagliptin. Therefore, the differentiation of macrophages and M2 polarization were significantly affected.

Taking together, the results of our study showed that in NSCLC, anagliptin enhanced the antitumor effect of PD-L1 blockade by targeting macrophage differentiation and M2 polarization. However, whether this effect could apply to tumors of different tissue origins and whether anagliptin directly affects the function of other kinds of immune cells in TME needed to be further explored.

## Conclusions

In summary, we demonstrated for the first time that the DPP4 inhibitor, anagliptin, plays an anti-tumor role as an ROS scavenger by inhibiting macrophage differentiation and M2 macrophage polarization, and it can potentiate the anti-tumor effect of PD-L1 blockade in NSCLC. This study provides a potential method and molecular mechanism for the combination therapy of DPP4 inhibitors with PD-L1 blockade. However, whether this effect applies to tumors of multiple tissue origins and whether other types of DPP4 inhibitors have the same effect need to be further confirmed.

### Supplementary Information

Below is the link to the electronic supplementary material.

Supplementary file1 (PDF 161 KB)

## Data Availability

The authors will provide data included in the present research upon request.
