# Supplementary material for: Dipeptidyl peptidase 4 inhibitor reduces tumor-associated macrophages and enhances anti-PD-L1-mediated tumor suppression in non-small cell lung cancer
Source: Clin Transl Oncol. 2023 Apr 28;25(11):3188–202. doi: 10.1007/s12094-023-03187-5 (PMC10514125; doi:10.1007/s12094-023-03187-5)
Supplement: Supplementary file 1 — Supplementary file1 (PDF 161 KB) [file 12094_2023_3187_MOESM1_ESM.pdf]

Supplementary appendix

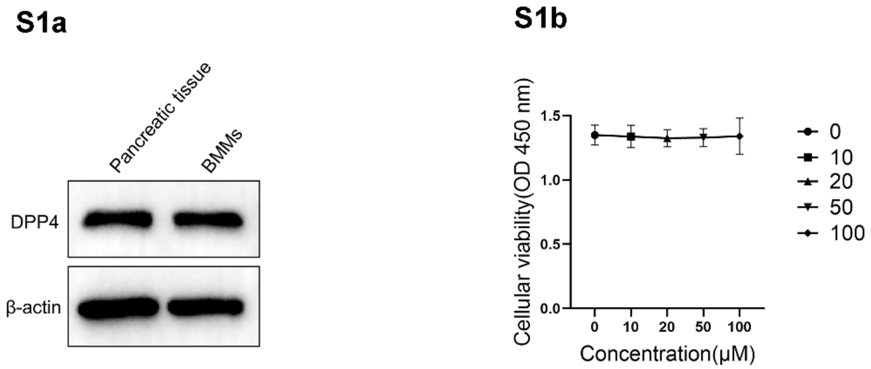

Supplementary Materials: **Fig. S1** a, Western blotting detected the expression of DPP4 on these monocytes, and pancreatic tissue was used as a positive control. b, CCK8 assay was used to observe the effects of different concentrations of anagliptin on the proliferation and viability of BMMs. Monocytes were either untreated or pretreated with different concentrations of anagliptin (10 μM, 20 μM, 50 μM, 100 μM) for 24 h, absorbance at 450 nm were detected and are presented (mean ± S.E)
